# Relating structure and function of inner hair cell ribbon synapses

**DOI:** 10.1007/s00441-014-2102-7

**Published:** 2015-01-22

**Authors:** C. Wichmann, T. Moser

**Affiliations:** 1 Molecular Architecture of Synapses Group, Institute for Auditory Neuroscience and InnerEarLab, University Medical Center Göttingen, Göttingen, Germany; 2 Collaborative Research Center 889, University Medical Center Göttingen, Göttingen, Germany; 3 Institute for Auditory Neuroscience and InnerEarLab, University Medical Center Göttingen, Göttingen, Germany; 4 Center for Nanoscale Microscopy and Molecular Physiology of the Brain, University of Göttingen, Göttingen, Germany; 5 Bernstein Center for Computational Neuroscience, University of Göttingen, Göttingen, Germany

**Keywords:** Structure–function, Inner hair cells, Ribbon synapse, Exo–endocytosis, Otoferlin

## Abstract

In the mammalian cochlea, sound is encoded at synapses between inner hair cells (IHCs) and type I spiral ganglion neurons (SGNs). Each SGN receives input from a single IHC ribbon-type active zone (AZ) and yet SGNs indefatigably spike up to hundreds of Hz to encode acoustic stimuli with submillisecond precision. Accumulating evidence indicates a highly specialized molecular composition and structure of the presynapse, adapted to suit these high functional demands. However, we are only beginning to understand key features such as stimulus–secretion coupling, exocytosis mechanisms, exo–endocytosis coupling, modes of endocytosis and vesicle reformation, as well as replenishment of the readily releasable pool. Relating structure and function has become an important avenue in addressing these points and has been applied to normal and genetically manipulated hair cell synapses. Here, we review some of the exciting new insights gained from recent studies of the molecular anatomy and physiology of IHC ribbon synapses.

## Introduction

Synapses transfer information from sensory cells or neurons to other neurons or distinct target cell types, such as muscle cells. A plethora of presynaptic proteins orchestrate neurotransmitter release at the presynaptic active zone (AZ). These proteins are organized into three main compartments, which are ultrastructurally defined and classically referred to as (1) the cytomatrix at the active zone (CAZ) with (2) presynaptic electron dense projections that are clustering (3) synaptic vesicles (Zhai and Bellen 2004). The presynaptic dense projections appear highly variable in size and shape, which have been hypothesized to follow the function of a given synapse type. They seem to be present at all neuronal AZs but differ greatly in terms of order, density and morphology as well as molecular composition (Zhai and Bellen 2004). For example, rather small structures of less than 100 nm height are found at mammalian conventional central nervous system (CNS) synapses where they form a presynaptic grid, also termed a ‘particle web’, with a triangular or hexagonal pattern (Vrensen et al. 1980; Phillips et al. 2001; Zhai and Bellen 2004; Limbach et al. 2011; Südhof 2012). Remarkably regularly arranged structures can be observed at neuromuscular junctions of the frog (Harlow et al. 2001; Szule et al. 2012). Moreover, presynaptic dense projections are not an evolutionary invention of vertebrates, as insects such as the fruitfly *Drosophila melanogaster* also harbor elaborated dense projections termed ‘T-bars’, which are found at almost every synapse type (for review, see Wichmann and Sigrist 2010). The anatomical hallmark of tonically releasing sensory mammalian photoreceptor synapses, a huge plate-like dense projection that tethers hundreds of synaptic vesicles (Schmitz et al. 2000), was discovered in the 1950s (De Robertis and Franchi 1956), when transmission electron microscopy started to become a commonly used technique.

Electron microscopy allowed researchers to visualize the ultrastructure of cells in detail for the first time (De Robertis and Bennett 1955), bringing exciting new knowledge about morphology, organization and communication of cells in general and synapses in particular (see, for example: De Robertis and Bennett 1955; De Robertis and Franchi 1956). At this time, synaptic vesicles were discovered at guinea pig retinal synapses, where they were called ‘minute granules’ (Sjostrand 1953). Soon afterwards, the term ‘synaptic vesicle’ was coined by De Robertis and Bennett (1955), who were inspecting bullfrog and earthworm synapses. In parallel, the work of De Robertis and Franchi (1956) on photoreceptors of light- or dark-exposed rabbits provided the first experimental evidence correlating synaptic vesicle numbers and presynaptic activity. A few years later, the large presynaptic dense structures of these synapses were named ‘ribbons’, when their characteristic shape with extended longitudinal axis was recognized in serial 3D reconstructions of guinea pig retinas (Sjostrand 1958). Subsequently, synaptic ribbons were also found to decorate cochlear afferent hair cell synapses (Smith and Sjostrand 1961).

Golgi or horseradish peroxidase labeling in combination with transmission electron microscopy were also and still are, widely used to visualize neurons (Meller et al. 1968; LeVay 1973; White and Rock 1980; DeFelipe et al. 1986) and to understand the anatomy of the inner ear. For example, the afferent spiral ganglion neurons (SGNs) of the cochlear nerve, which carry the information about an acoustical signal from the inner ear to the brainstem, were studied intensely in various mammals such as guinea pig, mouse or cat (Spoendlin 1972, 1975, 1979; Paradiesgarten and Spoendlin 1976; Bodian 1978; Kiang et al. 1982; Liberman 1982a; Ginzberg and Morest 1984; Ryugo and Rouiller 1988; Liberman et al. 1990). These studies revealed that inner and outer hair cells are innervated by different SGN types (Kiang et al. 1982), outer hair cells (OHCs) by unmyelinated (5 %) and inner hair cells (IHCs) by myelinated (95 %) afferent fibers (Spoendlin 1969, 1975). Each of the myelinated, bipolar type I SGNs sends a peripheral unmyelinated and unbranched neurite to form a synapse with a *single* IHC ribbon synapse (Liberman 1980; Liberman et al. 1990; Buran et al. 2010; reviewed in Meyer and Moser 2010). Therefore, recordings from SGNs enable the investigation of the function of individual AZs within an IHC. Type I SGNs show different intensity thresholds and dynamic ranges in cat (Liberman and Kiang 1978). Paired recordings from hair cells and postsynaptic neurons have provided insight into synaptic sound encoding and its presynaptic determinants (Palmer and Russell 1986). Finally, observations of postsynaptic excitatory potentials by recordings from near the synapse revealed the first information on the presynaptic release mechanism (Furukawa et al. 1978; Starr and Sewell 1991; Siegel 1992). Each IHC contains 5–30 AZs, dependent on species and tonotopic position along the cochlea, generally peaking at the region with the greatest sound sensitivity for the particular species (Francis et al. 2006; Meyer et al. 2009; Meyer and Moser 2010). Liberman and co-workers were among the pioneers coupling structural investigations of the mammalian auditory system to its function. In his seminal study, Liberman’s (1982b) functional characterization of cat single auditory nerve fibers was followed by horseradish peroxidase labeling to individually back-trace the innervation location at the respective IHC AZs. This approach allowed the author to relate functional parameters such as spontaneous firing rates and firing thresholds to morphology of type I SGNs, described, for example, by the dimension and location of their unmyelinated terminals on the IHCs. These studies together led to the hypothesis that ribbon synapses within a given IHC are structurally and functionally heterogeneous (which will be discussed later in this review) and pointed to the further need for detailed structure–function analyses. Horseradish peroxidase labeling combined with electron microscopy also provided insights into presynaptic vesicle cycling in hair cells (Siegel and Brownell 1986). More recently, hair cell synapses have increasingly attracted research activity and novel as well as classical methods have been employed for assessing their structure and function in combination with genetic or pharmacological manipulation of the synapses or noise exposure. Quantitative electron microscopy analysis employing electron tomography of different functional states as well as freeze-fracture and subsequent electron microscopy have been introduced by Roberts and others for studies of hair cell synapses (Roberts et al. 1990; Saito 1990; Lenzi et al. 1999, 2002). Molecular manipulations involving germline mutagenesis as well as virus-mediated gene transfer were established. Further, patch-clamp recordings have characterized Ca^2+^ currents (e.g., Lewis and Hudspeth 1983; Fuchs et al. 1990; Roberts et al. 1990; Platzer et al. 2000; Brandt et al. 2003) and membrane turnover (e.g., Parsons et al. 1994; Moser and Beutner 2000; Schnee et al. 2005) of hair cells. Technically very challenging postsynaptic patch-clamp recordings have provided insight into the excitatory postsynaptic currents (Glowatzki and Fuchs 2002) and, combined with presynaptic recordings, have elucidated hair cell synaptic mechanisms with superb resolution (e.g., Keen and Hudspeth 2006; Goutman and Glowatzki 2007; Li et al. 2009). Immunohistochemistry combined with high-resolution microscopy as well as transcriptomic and proteomic analyses have informed on the molecular composition of hair cell synapses (Khimich et al. 2005; Uthaiah and Hudspeth 2010; Kantardzhieva et al. 2011). Finally, fluorescence imaging has been implemented for studies of hair cell synapse function (Tucker and Fettiplace 1995; Issa and Hudspeth 1996; Zenisek et al. 2003; Griesinger et al. 2005; Frank et al. 2009; Revelo et al. 2014).

Ribbon-type AZs cope with a demanding task: synaptic vesicles need to be released indefatigably and rapidly recycled at individual synapses in order to maintain high firing rates of SGNs that fire at hundreds of Hz even during continued stimulation (reviewed in Matthews and Fuchs 2010; Pangršič et al. 2012; Safieddine et al. 2012). Sustained exocytosis amounts to up to 70 Hz from each release site, of which about a dozen comprise the readily-releasable vesicle pool (RRP). This was demonstrated in mouse IHCs (Pangršič et al. 2010) and is to our knowledge one of the highest release rates per site described to date (Pangršič et al. 2012). This process requires very efficient means of clearing previously exocytosed membrane and proteins from the site followed by immobilization and priming of new vesicles for the next round of release. Moreover, the release of the neurotransmitter must exhibit both rapid ON and OFF kinetics to accurately follow acoustic stimuli with a periodicity of 1 ms or less (Kiang et al. 1965; Rose et al. 1967; Palmer and Russell 1986; Köppl 1997; Goutman 2012; Li et al. 2014).

How the molecular machinery of IHC AZs meets these requirements is just starting to emerge. It is becoming clear that ultrastructural assessment of functional synapse states is required in addition to the powerful combination of molecular manipulation and physiological characterization. In this review, we will emphasize recent approaches coupling functional and structural investigations of release at the level of IHCs and their ribbon synapses, as well as recent findings regarding vesicular recycling after transmitter release.

### The structure of the inner hair cell is set up for efficient signaling

How does the subcellular organization of sensory IHCs enable mechanotransduction and transmitter release at high rates? IHCs are epithelial cells by origin and exhibit several characteristics that distinguish them from neurons. Most notably, they show a strong polarization with respect to both long and short cell axes. The polarization along the apicobasal axis follows a clear compartmentalization, e.g., apparent by the hair bundle harboring the mechanotransduction apparatus of the apical membrane. Graded receptor potentials are formed by mechanoelectrical (apical) and voltage-gated (basal) conductances (Corey and Hudspeth 1979; Roberts et al. 1990). Actin-filled stereocilia protrude into the endolymph in a highly organized manner and their sophisticated supramolecular mechanotransduction apparatus enables ultrasensitive detection of sound-born vibrations of the cochlear partition (reviewed in Kazmierczak and Müller 2012). While the molecular identity of the mechanotransducer channel still awaits definitive demonstration, recent work indicates the transmembrane channel-like proteins (TMC)-1 and -2 as promising candidates (Pan et al. 2013). Opening of the apical mechanotransducer channels depolarizes the IHC, subsequently activating Ca_V_1.3 Ca^2+^ channels (Platzer et al. 2000; Brandt et al. 2003; Dou et al. 2004) at the presynaptic AZ in the basolateral membrane, where the incoming Ca^2+^ triggers neurotransmitter release. The density of ribbon synapses shows a strong basoapical gradient, with the supranuclear portion of the hair cell being devoid of AZs (Francis et al. 2004; Meyer et al. 2009). In the apex, the cuticular plate likely serves as an anchor for the stereociliar actin bundles, containing a rich protein network with cytoskeletal proteins such as actin, α-actinin and tropomyosin (Slepecky and Chamberlain 1985; Zine and Romand 1993). Moreover, the striated organelle, located underneath the cuticular plate, likely modulates the stereociliar actin bundles (Vranceanu et al. 2012). Microtubules are primarily found beneath the cuticular plate (Slepecky and Chamberlain 1985; Steyger et al. 1989; Furness et al. 1990) but appear connected to cytoskeletal proteins in the cuticular plate, for example via Acf7a (actin crosslinking family protein 7a), as suggested for zebrafish neuromast hair cells (Antonellis et al. 2014). Microtubule bundles are mainly organized in the apicobasal direction (Furness et al. 1990), providing the mechanical strength of hair cells (Szarama et al. 2012) and tracks for efficient cargo protein trafficking along the apicobasal axis (Furness et al. 1990).

In addition to the cellular apicobasal polarity, hair cells also show planar cell polarity, which is reflected in the orderly orientation of their hair bundles (reviewed in Ezan and Montcouquiol 2013; Sienknecht et al. 2014). Whether the basolateral organization of the hair cells is similarly instructed by planar cell polarity remains to be tested.

In the next sections, we will focus on the organization of the basal portion of IHCs and discuss structure and function of hair cell ribbon synapses. Emphasis will be on the molecular machinery of the synapse, synapse development, synaptic heterogeneity and synaptic vesicle recycling.

### Molecular anatomy and physiology of hair cell ribbon synapses

**Fig. 1:**
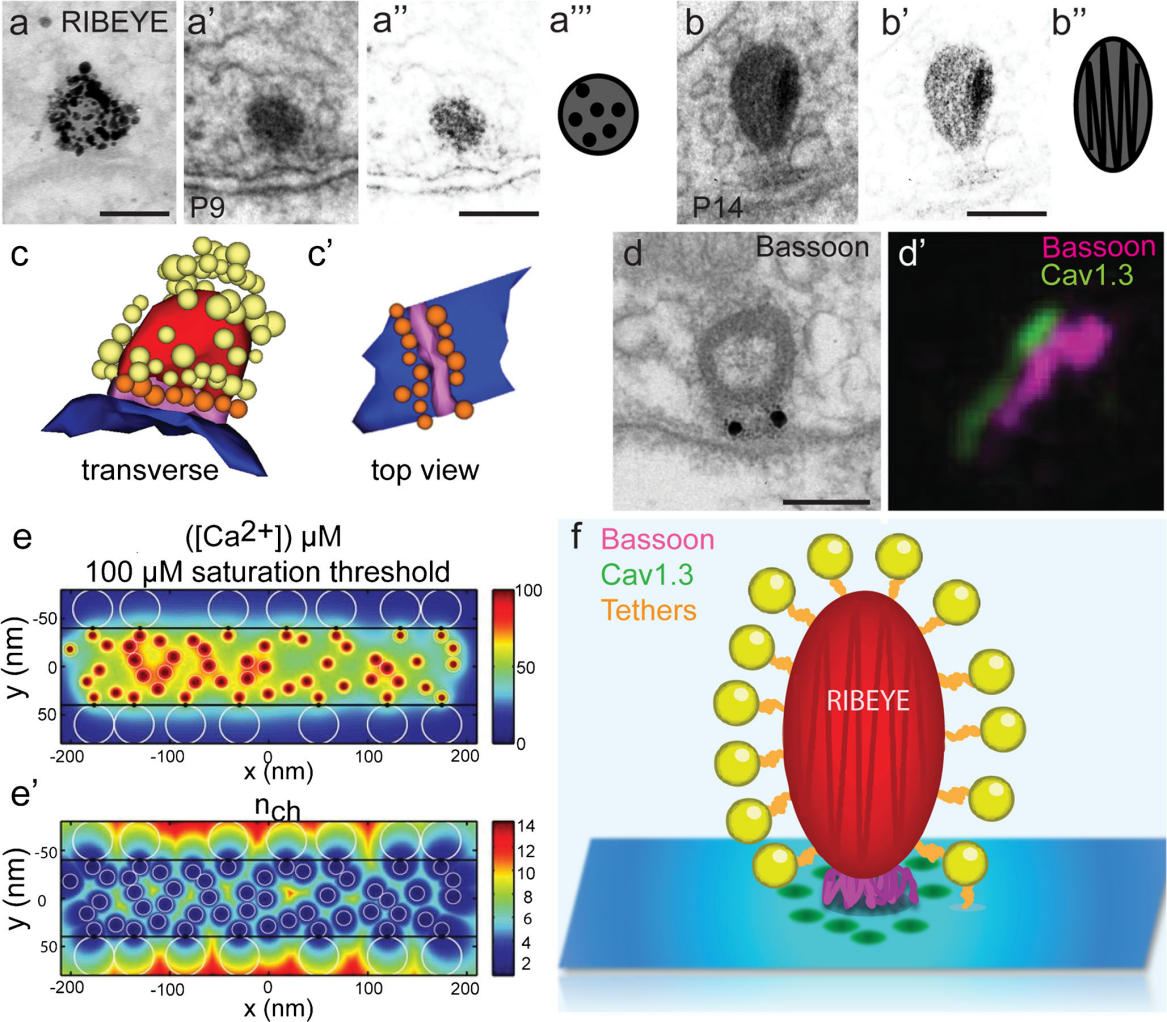
Spatial distribution of IHC AZ proteins. **a** RIBEYE is the main component of the ribbon as shown by pre-embedding immunogold labeling of a P14 IHC synaptic ribbon using an anti-CtBP2 antibody (courtesy of Susann Michanski, InnerEarLab, University Medical Center, Göttingen, Germany); **a’** Representative image of an electron micrograph of a round-shaped P9 immature ribbon exhibiting a dotted pattern possibly caused by RIBEYE arrangement (contrast enhanced image in **a”**), see also schematic representation (**a”’**). **b** A P14 mature ribbon with the typical multi-lamellar pattern (contrast enhanced image in **b’**), see also scheme in **b”**. *Scale bars* (**a**, **a”**, **b’**) 100 nm. **c** A serial 3D reconstruction of a mature ribbon with two distinct morphological vesicle pools (*yellow*: ribbon-associated vesicles; *orange*: membrane-proximal vesicles; *red*: ribbon; *blue*: AZ membrane; *magenta*: presynaptic density). **c’** The membrane-proximal vesicles (*orange*) are arranged around the presynaptic density (*magenta*) that is containing the scaffolding protein bassoon as shown by the pre-embedding immunogold labeling in (**d**), *Scale bar* (**d**) 100 nm (courtesy of Susann Michanski, InnerEarLab, University Medical Center, Göttingen, Germany); **d’** 2-color STED image of immunolabeled bassoon (*magenta*) and Ca_V_1.3 channel clusters (*green*) in mature IHCs: stripe‐like morphology and closely aligned immunofluorescence of bassoon and Ca_V_1.3 can be observed. *Scale image:*700 × 700 nm; **e**, **e’** Mathematic model showing the total mean steady state [Ca^2+^] profile at the AZ membrane (**e**); **e’** effective number of Ca_V_1.3 channels contributing to total mean steady state [Ca^2+^] as shown in (**e**). (**c**, **c’**, **d’**, **e**, **e’** modified from Wong et al. 2014, EMBO J; reprinted with permission © 2014 Wong et al.). **f** Schematic summary of the protein arrangement at mature IHC ribbon synapses

Phylogenetically, ribbons in sensory cells are old structures that occur not only in mammals but also in fishes, amphibians and birds. In the mammalian organ of Corti, they were first described by Smith and Sjöstrand (1961) and are found in both sensory cell types, i.e., IHCs and OHCs (Sobkowicz et al. 1982). The discovery of the protein RIBEYE, initially purified from bovine retina, (Schmitz et al. 2000), as the main and structure-yielding component of ribbons in rat photoreceptors (Schmitz et al. 2000), frog saccular hair cells (Zenisek et al. 2003), zebrafish photoreceptors and bipolar cells (Wan et al. 2005) and mouse cochlear hair cells (Khimich et al. 2005; see also immunogold labeling in Fig. 1a) highlights the conservation of the ribbon in vertebrate evolution (Schmitz 2009). Nonetheless, ribbons still vary greatly in size and shape (Lenzi and von Gersdorff 2001; Moser et al. 2006; Matthews and Fuchs 2010), likely reflecting structural adaptation to the specific needs of the respective synaptic connection for sensory coding.

RIBEYE is composed of two major domains: while the A domain organizes the assembly of the synaptic ribbon and is unique in structure, the B domain is structurally nearly identical to the transcription repressor CtBP2, which is encoded by the same gene but uses a different transcription initiation site (Schmitz et al. 2000) and exhibits enzymatic activity (Schwarz et al. 2011). The B domain is also assumed to be involved in tethering of synaptic vesicles to the ribbon (Schmitz et al. 2000; Schmitz 2009), though the proteins that form tethers remain to be identified. RIBEYE appears to organize ribbon shape directly based on its domain structure (Schmitz 2009) and its aggregation properties (Magupalli et al. 2008). A trifold lamellar pattern has been described for photoreceptor ribbons and assigned to the polarized arrangement of RIBEYE (Schmitz 2009). Also at mature IHC ribbons, a lamellar substructure is observed that harbors multiple lamellar foldings (Sobkowicz et al. 1982; Rutherford and Pangršič 2012; Fig. 1b–b”) contrasting photoreceptor ribbons. This effect can be attributed to the differences in the ribbon shape in IHCs and photoreceptors. In immature IHC ribbons, the lamellar pattern is not prominent but instead a dotted pattern can be observed (Fig. 1a’–a”’). Recently, at zebrafish hair cell ribbon synapses, it was found that the RIBEYE A and B domain segregate along the vertical axis of the ribbons, with the B domain more located towards the basal end (Sheets et al. 2014).

RIBEYE, in contrast to other AZ proteins, is not found in invertebrates such as the fruitfly *Drosophila melanogaster*; however, Bruchpilot (Brp), the homolog of the vertebrate AZ protein CAST (CAZ-associated structural protein)/ERC2, functions as the main building block of T-bars (Kittel et al. 2006; Wagh et al. 2006). In fact, only the N-terminus is conserved and shows sequence homologies to CAST, whereas the C-terminus is only found in dipteran insects and rather resembles cytoskeletal elements such as plectin due to its numerous coiled-coil domains (Wagh et al. 2006). Moreover, the C-terminus mediates vesicle tethering to the T-bar (Hallermann et al. 2010).

At conventional synapses of vertebrate neurons, the structurally related proteins bassoon and piccolo as well as CAST, ELKS, rab3-interacting molecule (RIM) and Munc13 are present (Betz et al. 1998; Fenster et al. 2000; Dresbach et al. 2001; Deguchi-Tawarada et al. 2006; Wang et al. 2009; Südhof 2012). Additionally, CtBP2 and CtBP1 have also been found at conventional AZs (tom Dieck et al. 2005). Except for piccolo, which at ribbon synapses is solely expressed as a shorter splice variant nicknamed as piccolino (Regus-Leidig et al. 2013), these proteins also largely form the CAZ at photoreceptor ribbon synapses (Wang et al. 1997; tom Dieck et al. 2005; Uthaiah and Hudspeth 2010; Limbach et al. 2011; Cooper et al. 2012). The components of hair cell AZs, on the other hand, are still largely unexplored, except for bassoon (Khimich et al. 2005) and piccolo/piccolino (Khimich et al. 2005; Regus-Leidig et al. 2013). In fact, a recent study indicates that IHC synapses operate without Munc13-like priming factors (Vogl et al. 2015).

Bassoon, together with RIBEYE, is responsible for the ribbon shape and, hence, might contribute to its function. Studies of IHCs from bassoon mutant mice indicated an anchoring function of bassoon (Khimich et al. 2005; Frank et al. 2010; Jing et al. 2013), in line with findings at photoreceptor ribbon synapses (Dick et al. 2003; tom Dieck et al. 2005). The fraction of ribbon-occupied synapses remaining in bassoon-deficient IHCs seems to depend on age and residual levels of full-length bassoon (Khimich et al. 2005; Frank et al. 2010; Jing et al. 2013). In the partial deletion mutant *Bsn* ^*ΔEx4*/*5*^, the fraction of ribbonless synapses increased from 50 % at postnatal day 11 (P11) up to 88 % at P70. A lower and relatively constant fraction of 56 % ribbonless synapses was found in a gene-trap bassoon mutant (*Bsn* ^*gt*^) likely due to a weak residual synaptic expression of bassoon (Jing et al. 2013). However, the anchorage of the remaining ribbons seems impaired (Frank et al. 2010; Jing et al. 2013). In line with a better maintained AZ ultrastructure, *Bsn* ^*gt*^ animals exhibited a larger number of Ca^2+^ channels at IHC synapses compared to *Bsn* ^*ΔEx4*/*5*^ mice and also displayed an intermediate phenotype regarding sustained IHC exocytosis (Jing et al. 2013). In contrast, the size of the readily releasable vesicle pool (RRP) was strikingly reduced in both mutants. Moreover, single unit recordings of the SGNs show comparably severe defects in *Bsn* ^*gt*^ and *Bsn* ^*ΔEx4*/*5*^ mice, as both genotypes had impaired sound onset coding and lower evoked and spontaneous spike rates. Taken together, these results indicate that the remaining, loosely anchored ribbons might function inadequately (Jing et al. 2013). This further suggests that the mere presence of the ribbon, even with tethered vesicles, is not sufficient to maintain normal transmitter release and sustain the RRP at IHC ribbon synapses. Moreover, it seems that bassoon contributes to organizing the IHC AZ beyond anchoring the ribbon. This has been concluded from impaired clustering of Ca^2+^ channels at *Bsn* ^*ΔEx4*/*5*^ ribbon-occupied synapses (Frank et al. 2010) and indicates a potential direct contribution of bassoon in organizing the AZ (Frank et al. 2010; Hallermann and Silver 2013).

In contrast to conventional synapses, where often only the combined knockdown of bassoon and its homolog piccolo causes synaptic defects (Altrock et al. 2003; Leal-Ortiz et al. 2008; Mukherjee et al. 2010; Waites et al. 2011, 2013), these proteins seem not to act redundantly at ribbon synapses. As mentioned above, at hair cell and photoreceptor ribbons, only the short isoform of piccolo, piccolino, is expressed, which lacks a large C-terminal part (Regus-Leidig et al. 2013, 2014). Binding sites for the proteins Abp1, Pra1, GIT1 and profilin (Wang et al. 1999; Fenster et al. 2000, 2003; Kim et al. 2003) are still present, whereas binding sites for, e.g., bassoon or RIM are lacking (Regus-Leidig et al. 2013). Therefore, different functions of piccolino and bassoon at ribbons synapses can be assumed. Accordingly, piccolino exhibits a different spatial distribution at ribbon synapses of photoreceptors, where it is found directly on the ribbon, as indicated by pre-embedding immunogold-labelings, using an antibody recognizing the N-terminus of the protein (Limbach et al. 2011; Dick et al. 2001). Furthermore, a recent study by Regus-Leidig et al. (2014) revealed a striking impairment in the ribbon structure upon piccolino RNAi-based knockdown. After piccolino knockdown, the typical plate-like structure failed to form properly. Instead, a high proportion of attached spherical ribbons was found that resemble ribbon precursors of photoreceptor ribbons suggesting a role of piccolino in structural ribbon maturation (Regus-Leidig et al. 2014). Taken together, RIBEYE and presumably piccolino as well as bassoon present the main structural components of the ribbon and/or the anchorage of the ribbon to the AZ.

In order to resolve the function of ribbons, several hypotheses have been put forward. The ribbon was suggested to (1) promote a large readily releasable pool of vesicles via establishing/stabilizing many Ca^2+^ channels and vesicular release sites (Khimich et al. 2005; Frank et al. 2010), (2) facilitate vesicle replenishment at the AZ (conveyor belt model, e.g., Bunt 1971; Gray and Pease 1971; Vollrath and Spiwoks-Becker 1996;  Lenzi and von Gersdorff 2001; Snellman et al. 2011), (3) facilitate multivesicular release (Edmonds 2004; Fuchs 2005), or (4) serve as a diffusion barrier to enable high local Ca^2+^ concentrations (Graydon et al. 2011). Functional interpretations have also been provided for the morphologically distinct populations of synaptic vesicles at ribbon synapses but in each case remain to be validated. Ribbon-associated vesicles—structurally attached through filamentous protein tethers—form a halo around the ribbon (Fig. 1b, c). The ones at the base of the ribbon face the presynaptic plasma membrane (membrane-proximal vesicles; Fig. 1c, c’) and are often tethered to the membrane and/or to the presynaptic density (tethered vesicles). Lateral to this subpopulation of ribbon-associated vesicles, there are few additional membrane-proximal and even membrane-tethered vesicles that are not in close vicinity to the ribbon. While further testing is required, current evidence suggests that the membrane-proximal vesicles comprise the RRP (e.g., in retinal bipolar cells: von Gersdorff et al. 1996; Zenisek et al. 2000; frog saccular hair cells: Lenzi et al. 1999, 2002; Rutherford and Roberts 2006; and mouse inner hair cells: Khimich et al. 2005; Frank et al. 2010; Wong et al. 2014). Support for this hypothesis comes from the approximate matching between the morphologically estimated number of membrane-proximal vesicles and the functionally defined size of the RRP, i.e., the fast component of exocytosis upon depolarization-evoked Ca^2+^ influx (von Gersdorff et al. 1996; Pangršič et al. 2010; Frank et al. 2010), as well as from the observation that these vesicles are most heavily depleted upon stimulation (Lenzi et al. 2002; Pangršič et al. 2010). Furthermore, the tethering of vesicles to the AZ membrane might reflect a structural correlate of fusion competence (Siksou et al. 2009; Frank et al. 2010; Fernández-Busnadiego et al. 2013). New high-resolution imaging approaches using rapid freezing methods and/or electron tomography have revealed that synaptic vesicles in proximity to the membrane exhibit several morphologically distinct stages. Tethers of different numbers and lengths connecting synaptic vesicles to the AZ membrane could be observed at conventional synapses and synaptosome preparations (Siksou et al. 2007, 2009; Fernández-Busnadiego et al. 2010, 2013) but also at IHC ribbon synapses (Frank et al. 2010). Moreover, cryo-electron tomography, a method allowing visualization of hydrated and unstained tissue, revealed that tethering of synaptic vesicles in synaptosomes prepared from hippocampal tissue precedes the full contact of a synaptic vesicle with the membrane (Fernández-Busnadiego et al. 2010). In this process, single long tethers initially seem to be formed and synaptic vesicles likely enter the RRP via the formation of several short tethers (<5 nm). In line with this hypothesis, this fraction of vesicles could be depleted by application of hypertonic sucrose, that is thought to trigger RRP release (Rosenmund and Stevens 1996). Moreover, the formation of short tethers could be inhibited using tetanus toxin, pointing towards the fact that neuronal soluble NSF attachment protein receptors (SNARE) proteins are involved in this process (Fernández-Busnadiego et al. 2010). The priming factors Munc13-1 (Betz et al. 2001; Siksou et al. 2009; Fernández-Busnadiego et al. 2013) and RIM1α (Fernández-Busnadiego et al. 2013) play a crucial role in tethering vesicles to the membrane at conventional CNS synapses. Interestingly, Munc13 and CAPS priming factors seem not to operate at the IHC ribbon synapse (Vogl et al. 2015). Some authors even argue that the entire ribbon-associated vesicle population is fusion-competent and, therefore, can be released within a few milliseconds or less (Heidelberger et al. 1994; Edmonds 2004). Evidence for a priming function of the ribbon has recently been presented (Snellman et al. 2011).

In contrast to CAZ proteins, which are, at least in part, conserved at IHC AZs, the molecular machinery involved in the regulation of synaptic vesicle fusion seems to deviate strongly from that of ‘conventional’ CNS synapses.

As mentioned above, neurotransmitter release at conventional synapses is mediated by neuronal SNAREs, namely SNAP-25, syntaxin 1 and synaptobrevin 1 or 2 (reviewed in Jahn and Fasshauer 2012). SNARE activity can be blocked by neurotoxin-mediated cleavage or genetic manipulations (Schiavo et al. 1992, 2000; Nouvian et al. 2011). At photoreceptor ribbon synapses, the SNARE protein machinery appears to be present and functional (Brandstätter et al. 1996; Morgans et al. 1996; Morgans 2000; von Kriegstein and Schmitz 2003; Uthaiah and Hudspeth 2010; Cooper et al. 2012). In contrast, IHC exocytosis seems to be insensitive to neurotoxins and genetic ablation of neuronal SNAREs (Nouvian et al. 2011) and, hence, a functional role of syntaxin 1, synaptobrevin 1 and 2 as well as SNAP-25 in IHC exocytosis is questionable. While some studies detected SNARE mRNAs and proteins in the sensory epithelium and hair cells (Safieddine and Wenthold 1999; Uthaiah and Hudspeth 2010; Nouvian et al. 2011), neither SNAP-25, synaptobrevin 1–3 nor syntaxin 1 could be detected by immunofluorescence imaging in mouse IHCs (Nouvian et al. 2011). Moreover, SNARE regulators such as synaptotagmins 1–3 (Beurg et al. 2010; Reisinger et al. 2011) and complexins (Strenzke et al. 2009; Uthaiah and Hudspeth 2010) appear to be absent from mature IHCs. In contrast, the multi-C_2_ domain protein otoferlin plays a central role for hair cell exocytosis (Roux et al. 2006; Beurg et al. 2008; Dulon et al. 2009; Pangršič et al. 2010).

The absence or mutation of otoferlin causes deafness or temperature-sensitive hearing impairment in humans (Yasunaga et al. 1999; Varga et al. 2006; Rodríguez-Ballesteros et al. 2008) and rodents (Roux et al. 2006; Longo-Guess et al. 2007; Schwander et al. 2007). Ultrastructurally, otoferlin is also found at ribbon synapses, mostly but not exclusively at synaptic vesicles and the AZ membrane (Roux et al. 2006). Sufficient amounts of otoferlin appear to be required for correct vesicular fusion and replenishment (Roux et al. 2006; Pangršič et al. 2010, 2012). Otoferlin is suggested to act as the Ca^2+^ sensor in IHCs (Roux et al. 2006; Johnson and Chapman 2010), due to its Ca^2+^-binding capabilities (Roux et al. 2006; Ramakrishnan et al. 2009, 2014; Johnson and Chapman 2010; Pangršič et al. 2010) and facilitates SNARE-mediated liposome fusion (Johnson and Chapman 2010). In its absence, no depolarization-evoked RRP exocytosis is observed in IHCs (Roux et al. 2006; Pangršič et al. 2010). Transgenic expression of synaptotagmin 1, the major Ca^2+^ sensor of neuronal synaptic vesicle exocytosis, failed to restore IHC exocytosis and hearing in otoferlin KO mice, which may not be too surprising given the overall low conservation of the molecular composition between conventional and IHC synapses (Reisinger et al. 2011).

Next to proteinaceous exocytosis machineries, the actual mechanisms of vesicle fusion as well as the transport of vesicles to the IHC release site are still largely unknown. The large and, in terms of amplitude and shape, heterogeneous excitatory postsynaptic currents (EPSCs) measured at postsynaptic afferent boutons of SGNs have been interpreted to result from multivesicular (multiquantal) release at IHC AZs (Glowatzki and Fuchs 2002). Large EPSCs ensure rapid and temporal precise spike generation of SGNs (Rutherford et al. 2012) and the relevance of such large EPSCs for achieving a high synchronization index of postsynaptic firing (i.e., better phase locking precision) has recently been shown in the frog papilla (Li et al. 2014).

Several multiquantal release scenarios at IHC ribbons have been discussed: (1) synchronized vesicle fusion of several single vesicles as well as (2) compound fusion, following homotypic vesicle-to-vesicle fusion and (3) sequential fusion involving homotypic vesicle-to-vesicle fusion while release occurs (Glowatzki and Fuchs 2002; Edmonds 2004; Neef et al. 2007). Recent findings suggest an alternative candidate mechanism for IHC exocytosis. Combining experimental approaches and mathematical modeling Chapochnikov et al. (2014) indicated that univesicular (uniquantal) release can explain the large size of SGN EPSCs and that the control of release by a dynamic vesicular fusion pore can account for the observed EPSC heterogeneity. At this point, none of the above discussed mechanisms can definitively be ruled out or confirmed and future work, including detailed morphological analysis using electron microscopy of defined functional states, will be required to advance our understanding of exocytosis mechanism at IHC ribbon synapses.

In case vesicles do not homotypically fuse with other vesicles at the ribbon while releasing, a transport mechanism of the vesicles to the membrane has to exist. The conveyor belt model, transporting the vesicle actively along the ribbon to the membrane, was one of the first models to be introduced (Bunt 1971; Gray and Pease 1971; Vollrath and Spiwoks-Becker 1996; Lenzi and von Gersdorff 2001). Accordingly, a kinesin polypeptide, Kif3a, was identified on photoreceptor ribbons that could serve as a motor for vesicle transport involving the filamentous tethers observed at the ribbon (Muresan et al. 1999), which were also proposed to function as ‘stepping stones’ for synaptic vesicles (Usukura and Yamada 1987; Parsons and Sterling 2003). Recently, the tethers at the ribbon were suggested to be directly involved in coordinating vesicle transport towards the membrane via ‘crowd surfing’, based on passive diffusion following the gradient established by exocytic vesicle consumption at the base of the ribbon (Graydon et al. 2014). In this model, the tethers simply need to bind the vesicles and prevent them from detaching until they reach the AZ membrane, where release maintains the diffusion gradient (Graydon et al. 2014). However, future experiments involving mutant analyses will be necessary to identify the proteins mediating vesicular tethering to the ribbon and estimate their affinity to the vesicles and the functional relevance of the tethers for synaptic transmission. Moreover, it will be interesting to investigate whether and how the tethering can be influenced by factors such as activity or even developmental stage. For example, maturation from pre-hearing to hearing significantly determines structure and function of the ribbon synapses and the spatial arrangement of AZ proteins such as the Ca^2+^ channels or bassoon, as will be emphasized in the next section.

### Structural and functional maturation of inner hair cell ribbon synapses

During maturation of the organ of Corti, ribbon synapses and SGN fibers undergo drastic morphological changes. How do morphological alterations during the transition from a pre-hearing to a hearing state correlate to functional maturation of ribbon synapses? Generally, synaptic contacts are ultrastructurally defined as pre- and postsynaptic electron-dense membranes that are closely aligned. The postsynaptic density (PSD) is clearly visible as an electron-dense structure beneath the postsynaptic membranes directly juxtaposed to the presynaptic AZ. The innervation pattern of SGN fibers at hair cells within the immature rodent cochlea is significantly different from the mature configuration and massive rearrangements of the fibers that occur before the onset of hearing. Hereby, type I SGN fibers retract from the OHCs, whereas type II SGN fibers disappear from the IHCs (Perkins and Morest 1975; Echteler 1992; Simmons 2002). These developmental processes of fiber innervation take place in the first postnatal week in three distinct phases: (1) in E18-P0 animals, fibers of both afferent types extend towards all hair cells; (2) between P0 and P3 a refinement occurs, where the outer spiral bundle forms that innervate the OHCs; and (3) the type I fibers retract from the OHCs around P3–P6, accompanied by synaptic pruning, while they keep their projections on the IHCs (Huang et al. 2007). In line with SGN fiber type I retraction, AMPA-typed glutamate receptors and scaffold proteins like bassoon and shank1 disappear during the maturation process from OHCs. In contrast, at IHC afferent PSDs AMPA-receptors persist. GluA2/3 subunits remain stable throughout development and into adulthood, while GluA4 subunit expression significantly increase in adult type I fibers (Huang et al. 2012).

Recently, the molecular arrangement of afferent synapses in relation to functional changes at the IHCs has been addressed in more detail using a combination of confocal, stimulated emission depletion (STED) and electron microscopy, as well as IHC presynaptic physiology and computational modeling (Wong et al. 2014). It is known that, in the early pre-hearing stages between P6 and P9, several small apposing pre- and postsynaptic densities mark nascent synapses. Some of the presynaptic densities are occupied by synaptic ribbons, which are small and round in shape and attached via two triangular-shaped proteinaceous anchors (Sobkowicz et al. 1982; Wong et al. 2014). However, floating ribbons were also frequently observed in close proximity to AZ areas at these developmental stages (Wong et al. 2014). Serial 3D electron microscopic reconstructions corroborated the notion of several discontinuous pre- and postsynaptic specializations. Such synaptic sites are organized as loose suprastructures on the bouton surface and are likely functional, as immunohistochemistry indicates the presence of presynaptic Ca^2+^ channels and postsynaptic AMPA receptors (Wong et al. 2014). STED microscopy, which enables resolution below the diffraction limit (Klar et al. 2000; Hell 2007), revealed that Ca_V_1.3 channels are arranged in small round spots (Wong et al. 2014) rather than the stripes previously described for mature AZs (Frank et al. 2010; see also Fig. 1d’). In addition, a huge number of extrasynaptic Ca_V_1.3 channels can be observed in immature IHCs (Zampini et al. 2010; Wong et al. 2014), which enable the cells to fire Ca^2+^ action potentials (Kros et al. 1998; Brandt et al. 2003). These action potentials evoke exocytosis in the pre-hearing stage (Beutner and Moser 2001; Glowatzki and Fuchs 2002; Johnson et al. 2005) but show lower ‘Ca^2+^ efficiency’ (Beutner and Moser 2001; Brandt et al. 2003; Johnson et al. 2005) and a supra-linear Ca^2+^ dependence (Johnson et al. 2005). The pre-sensory IHC activity appears to drive bursting activity in the developing auditory system (Glowatzki and Fuchs 2002; Tritsch et al. 2007, 2010; Wong et al. 2013; Clause et al. 2014). In this context, the regulation of presynaptic firing by paracrine and/or efferent synaptic control is being subject to intense research (Glowatzki and Fuchs 2000; Tritsch et al. 2007; Johnson et al. 2011; Sendin et al. 2014). Efferent innervation, moreover, seems to play an important role in the maturation process of IHCs (Glowatzki and Fuchs 2000; Marcotti 2004; Goutman et al. 2005). Efferent fibers originate from the superior olivary complex and, before onset of hearing, form transient axosomatic contacts with IHCs (Simmons et al. 1996; Katz et al. 2004). Later, they largely retract from IHCs and rather form axodendritic contacts to the afferent terminals (Pujol et al. 1998). The transient efferent inhibition is thought to counteract the IHC depolarization resulting from the resting mechanotransducer current (Géléoc and Holt 2003; Waguespack et al. 2007; Lelli et al. 2009). Upon genetically induced impairment of the efferent input, the linearization of Ca^2+^ dependent exocytosis is affected (Johnson et al. 2007) and the maturation of IHC afferent synapses is also disturbed (Johnson et al. 2013b). Around the onset of hearing (at around P11; Mikaelian and Ruben 1965), when graded receptor potentials start governing transmitter release, extrasynaptic Ca_V_1.3 channels get pruned and spatial coupling of Ca^2+^ channels and vesicular release sites is tightened. This leads to an increase of the ‘Ca^2+^ efficiency’ of exocytosis and a near-linear Ca^2+^ dependence of RRP exocytosis when probed with changes in the number of open Ca^2+^ channels (Wong et al. 2014). Therefore, while the intrinsic Ca^2+^ dependence of exocytosis apparently does not change upon the onset of hearing, experimental data and biophysical modeling of exocytosis at mature and immature AZ topographies support the notion of a developmental switch from the more ‘Ca^2+^ microdomain-like control’ of exocytosis by several Ca^2+^ channels per vesicle to a more ‘Ca^2+^ nanodomain-like control of exocytosis’ (Wong et al. 2014; Fig. 1e, e’). Interestingly, in adult gerbils, the open probability of Ca^2+^ channels in IHCs increased due to a preference of the Ca^2+^ channel for the bursting mode (Zampini et al. 2013).

Structurally, alongside Ca^2+^ channels, other presynaptic AZ components become reorganized such as the bassoon containing presynaptic density (Fig. 1d). These alterations are accompanied by changes of the postsynaptic glutamate receptor fields that also develop to one continuous ring-like cluster (Wong et al. 2014). Moreover, ribbons increase in size and undergo striking changes of shape. At the ultrastructural level, their cross-sectional shape changes from predominantly round (Fig. 1a’) to a rather oval-, droplet- or wedge-like shape between P14 and P20 (Wong et al. 2014; Fig. 1b) and ribbon architecture extends in the longitudinal direction (Sobkowicz et al. 1982; Wong et al. 2014) likely by gaining additional ribbon material. The two rootlets seem to merge to a continuous presynaptic density that contains the scaffolding protein bassoon, as revealed by immunogold labeling (Wong et al. 2014; Fig. 1d). Shortly after onset of hearing at P14, a large proportion of ribbons with two rootlets can still be found, whereas about a week later the morphological maturation appears to be completed (Wong et al. 2014, see, for summary, Fig. 1f). Factors that participate in the maturation of IHC synapses, next to the efferent olivocochlear transmission (see above; Johnson et al. 2013a), are thyroid hormone (Sendin et al. 2007) and myosin 6 (Heidrych et al. 2009; Roux et al. 2009). For both, a higher proportion of morphological immature ribbons have been observed in genetic deletion models.

In conclusion, during development from pre-hearing to hearing, IHC ribbon synapses undergo major morphological and functional refinements, resulting in tighter spatial coupling between Ca^2+^ influx and exocytosis (Wong et al. 2014).

### Dynamics and heterogeneity of hair cell ribbon synapses

The number of Ca^2+^ channels, vesicular release sites and ribbon-associated vesicles seems to scale with the size and number of ribbons at the AZ (Martinez-Dunst et al. 1997; Frank et al. 2009; Graydon et al. 2011; Kantardzhieva et al. 2013; Wong et al. 2013, 2014). Strengthening of presynaptic transmitter release might therefore be accomplished by increasing ribbon or AZ size and/or ribbon numbers per AZ. Moreover, synaptic strength might be determined by the amount and distribution of postsynaptic AMPA receptors. Finally, lateral olivocochlear efferent fibers might modulate postsynaptic excitability and thereby affect afferent synaptic strength. To establish which of these mechanisms contribute to determining and regulating synaptic strength of hair cell synapses awaits further structural and functional characterization.

**Fig. 2:**
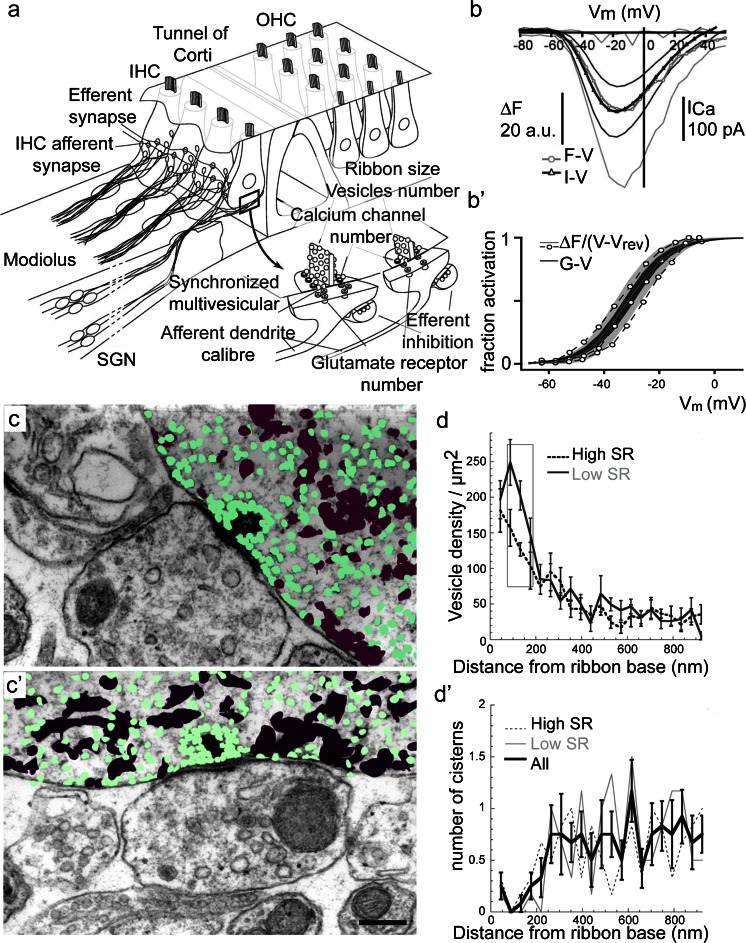
Principle of functional heterogeneity in IHCs. **a** Schematic of an organ of Corti showing afferent and efferent innervations at IHCs. Modified from Meyer and Moser 2010, Curr Opin Otolaryngol Head Neck Surg, reprinted with permission from © 2010 Wolters Kluwer Health. **b**, **b’** Heterogeneous Ca^2+^ signaling in IHCs. **b** Mean and SD of Δ*F* (*gray*) as a function of depolarizing potential (*V*_m_), obtained from spot-detection experiments at the center of the Ca^2+^ microdomain; Δ*F* was averaged over the last 15 ms of a 20-ms stimulus. Δ*F* (mean *gray*) and *I*_Ca_ (mean *black*) show a similar voltage dependence (*thin lines* corresponding SDs). **b’** Heterogeneous voltage dependence and Ca^2+^ channel number of synaptic Ca^2+^ channel clusters in IHCs. Pronounced variability in the voltage dependence of activation, even within the same cell (*dashed traces* individual data curves from 3 Ca^2+^ microdomains in an IHC). Modified from Frank et al. (2009), PNAS USA, with permission from © Frank et al. **c**, **c’** Colorized spatial distribution of vesicles and cisterns around the ribbon in low- and high-spontaneous rate (SR) fibers. Sections through a high-SR (**c**) and a low-SR (**c’**) synapse containing the synaptic ribbon are shown with cisternal (*maroon*) and vesicular (*green*) profiles. *Scale bar* (**c**m **c’**) 200 nm. **d**, **d’** Distribution of docked vesicles and cisterns. **d** Mean density (± SE) of docked vesicles, i.e., within 20 nm of the presynaptic density along the presynaptic membrane. **d’** Mean number (± SE) of cisterns within 20 nm of the presynaptic density versus distance along the presynaptic membrane is shown for all synapses. In addition, counts for low- and high-SR synapses are plotted separately. *Rectangle* the area of significant differences between low- and high-SR synapses. *SE* standard error; *SR* spontaneous rate. (**c**, **c’**, **d**, **d’** modified from Kantardzhieva et al. 2013, J Comp Neurol, reprinted with permission from © 2013 Wiley Periodicals)

Interestingly, the size and shape of ribbons appear to be highly variable and dynamic. In fact, in photoreceptors, these parameters strongly correlate with activity in light (silent) or dark (active) conditions (Spiwoks-Becker et al. 2013). Similarly, in IHCs, a diverse spectrum of ribbons has also been observed (Bodian 1978; Sobkowicz et al. 1982; Merchan-Perez and Liberman 1996; Wong et al. 2014). The specific ultrastructural properties seem to depend on several factors: (1) the maturation/age (see section above), (2) position within the inner hair cell and maybe also (3) dynamic adaptation to activity. A pioneering study in cats was one of the first to identify the correlation between structural heterogeneity of ribbon synapses and functional characteristics of auditory nerve fibers (Merchan-Perez and Liberman 1996). Surprisingly, large AZs with big and/or several ribbons, supposedly reflecting large presynaptic strength, seem to drive SGNs with low spontaneous rate and high thresholds (see also scheme in Fig. 2a). Whereas this conundrum remains unsolved, the mechanisms of functional presynaptic heterogeneity are now beginning to be understood. Evidence for such heterogeneity within individual IHCs was obtained using confocal imaging of presynaptic Ca^2+^ influx (Frank et al. 2009; see also Fig. 2b, b’). This study showed that presynaptic Ca^2+^ signals varied substantially in amplitude and voltage-dependence among the AZs within individual IHCs. The amplitude of the Ca^2+^ signal scaled with ribbon size as approximated by simultaneous imaging of a fluorescently tagged RIBEYE-binding peptide (Frank et al. 2009) and seemed to be greater at the neural side of the IHCs (Meyer et al. 2009). Linking such estimates to the functional and morphological properties of the postsynaptic neurons will be an important task for future studies. So far, correlative arguments based on coincidental changes in maximal strength of presynaptic Ca^2+^ influx and postsynaptic spiking during development and upon genetic disruption as well as modeling have been brought forward to argue that strong synapses drive SGNs that have high spontaneous rates and low thresholds (Wong et al. 2013). Interestingly, an inverse correlation of pre- and postsynaptic parameters of synaptic strength has recently been reported for mouse IHCs: Liberman et al. (2011) suggested that synapses with many AMPA receptors exhibit small ribbons. The authors favored the interpretation that the SGNs inserting at the neural (modiolar) face of IHCs exhibit low spontaneous rates and high thresholds despite their corresponding large IHC AZs, because they have a smaller complement of AMPA receptors than those at the neural (pillar) side. This would agree with the conclusion of the classical study, which showed a neural–abneural gradient of AZ size using electron microscopy for cat IHCs whereby large AZs faced SGNs with low spontaneous rates and high thresholds (Merchan-Perez and Liberman 1996). In a laborious approach, the authors traced 11 functionally-characterized fibers to the IHCs using serial 3D reconstructions of ultrathin sections. In this way, it was possible to directly correlate morphological parameters such as ribbon length, fiber contact area, synaptic plaque area and synaptic vesicle numbers to the functional parameters determined prior to fiber labeling using single unit recordings. Recently, such a gradient was also suggested for mouse IHCs and reported to be influenced by the lateral olivocochlear innervation (Yin et al. 2014). The segregation of nerve fibers on neural and abneural sides was further observed in a study investigating the abundance of mitochondria in postsynaptic terminals. Here, postsynaptic boutons facing the abneural side seem to harbor more mitochondria (Francis et al. 2004). Monitoring EPSCs from single afferent boutons, which is a suitable method to address synaptic function on the level of individual release sites (Glowatzki and Fuchs 2002), further showed differences among synapses. In these experiments, varying fractions of multiphasic EPSCs were observed and proposed to underlie the diverse firing properties of SGNs (Grant et al. 2010).

Further insights into the morphological heterogeneity require modern 3D reconstructions of larger volumes such as serial block face scanning electron microscopy (Denk and Horstmann 2004) or focused ion beam scanning electron microscopy (see, e.g., Knott et al. 2008; Kreshuk et al. 2011). Using these methods, a detailed 3D view of single IHCs including afferent and efferent innervations would be possible, finally allowing correlation of parameters, such as presynaptic ribbon size and postsynaptic bouton diameter, as a function of position on the IHC for a large number of synapses, together with the functional assessment of pre- and postsynaptic properties in cochlear explants by electrophysiology (Goutman and Glowatzki 2007) and/or imaging of IHC Ca^2+^ and exocytosis (e.g., Frank et al. 2009; Neef et al. 2014) and postsynaptic activity (Boyer et al. 2004).

### Synaptic vesicle recycling in inner hair cells

Tight coupling between exo- and endocytosis is a prerequisite for maintaining the enormous vesicle turnover rates at ribbon synapses. The underlying mechanisms of endocytosis in IHCs are just starting to become uncovered. Clathrin-coated structures but also large cisterns without clathrin-coats, are observed close to synaptic ribbons (Siegel and Brownell 1986; Sendin et al. 2007; Frank et al. 2010; Kantardzhieva et al. 2013; Neef et al. 2014; Revelo et al. 2014). Kantardzhieva et al. (2013) set out to determine whether such cisterns participate in vesicle reformation and what differences can be observed in correlation to the functional properties of high and low spontaneous rate fibers (Fig. 2c, c’, d, d’). An extensive quantitative analysis of ribbons, vesicles and cisterns from serial sections of cat IHC ribbon synapses suggested a ‘sphere of influence’ of 350 nm around the ribbon (Kantardzhieva et al. 2013). Here, fewer cisterns and more synaptic vesicles are found, which indeed points towards a contribution of cisterns to locally restricted vesicle formation. Other studies used membrane capacitance measurements to provide an initial functional assessment of endocytic membrane retrieval at IHC AZs (Moser and Beutner 2000; Beutner and Moser 2001; Neef et al. 2014). Moreover, pH-sensitive GFP (pHluorin; Miesenböck et al. 1998) targeted to the intraluminal face of vesicle membranes by attachment to vesicular glutamate transporters (Zhu et al. 2009) has become an important tool in studying exo- and endocytosis, not only from neurons but also IHCs (Neef et al. 2014; Revelo et al. 2014). Additionally, a novel membrane tracer specifically tailored to use in the organ of Corti has been devised and applied to investigate endocytosis (Revelo et al. 2014), as the commonly used styryl dye FM1-43 penetrates stereociliar mechanotransduction channels and hence is of limited use to study endocytosis, in IHCs (Gale et al. 2001; Kamin et al. 2014; Revelo et al. 2014). To date, expression analysis and immunohistochemistry have revealed the presence of several important molecular players of endocytosis such as dynamins, amphiphysin, clathrin (Neef et al. 2014) and adaptor protein 2 (AP-2) (Duncker et al. 2013) in IHCs. A very recent DNA microarray study investigating IHC and OHC transcriptomes might even give more insight into proteins involved in vesicle recycling (Liu et al. 2014).

**Fig. 3:**
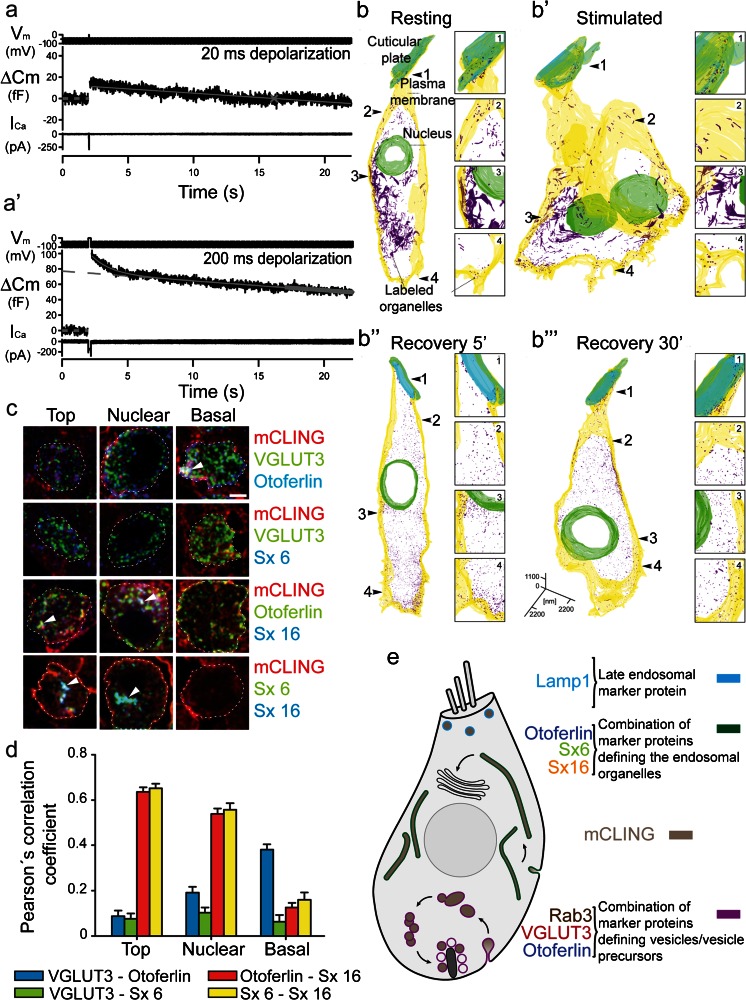
Endocytosis in inner hair cells. **a**, **a’** Representative recordings in response to 20 ms (**a**) or 200 ms (**a’**) depolarizations. After the *C*_m_ increase upon 20 ms depolarization, the slope-corrected *C*_m_ traces (*middle*) typically showed a linear decay (**a**). The 200-ms-long depolarization resulted in a combination of exponential and linear decay (**a’**). Modified from Neef et al. (2014) reprinted with permission from © 2014 Neef et al. **b**–**b”’** 3D reconstructions of resting (**b**), stimulated (**b’**) and recovered IHCs (**b”**, **b”’**). Endocytotic organelles are shown in *purple*. Note the presence of tubular organelles both before and after stimulation. Most organelles, including the tubular ones, are replaced by small vesicles during the recovery periods. *Insets* magnified regions from the four different cell regions (cuticular plate, top, nuclear and basal regions). Note the increased number of endosome-like organelles at the base of the cell after stimulation and during recovery. Modified from Kamin et al. (2014), reprinted with permission © 2014 Kamin et al. **c** mCLING-labeled organs of Corti were immunostained for Vglut3 and otoferlin (*first row*), for Vglut3 and syntaxin 6 (*Sx 6*, *second row*), for otoferlin and syntaxin 16 (*Sx 16*, *third row*) and finally for syntaxin 6 and syntaxin 16 (*fourth row*). The samples were cut into 20-nm sections and were imaged using an epifluorescence microscope. *Dashed white lines* the plasma membrane of the IHCs. *White arrowheads* organelles where the signals for mCLING and the two immunostained proteins colocalized. *Scale bar* 2 μm. **d** Graphic representation of Pearson’s correlation coefficients: otoferlin and syntaxin 6 (or syntaxin 16) correlate in the mCLING-labeled organelles at the top and nuclear levels. Vglut3 correlates best with otoferlin at the basal level. At least 100 organelles were analyzed for each condition. *Error bars* SEMs. **e** Model of membrane recycling in IHCs. Organelles with a different molecular composition recycle membrane in different regions, taking up mCLING. Apical endocytosis takes up the membrane into round organelles, a sizeable proportion of which is similar to late endosomes (*light blue*). Endocytosis in the top and nuclear regions reaches tubular organelles containing otoferlin and two endosome markers, syntaxin 16 and syntaxin 6. This suggests that these organelles participate in constitutive pathways, probably by maintaining membrane traffic between the plasma membrane and the trans-Golgi. At the base of the cell, stimulation induces the formation of membrane infoldings and cisterns that are characterized by the presence of Vglut3, Rab3 and also otoferlin. (**c**–**e** modified from Revelo et al. 2014, reprinted with permission from © 2014 Revelo et al.)

Currently, in IHCs, three distinct mechanisms are considered to mediate endocytosis: slow CME, fast bulk endocytosis and potentially kiss-and-run or ‘ultrafast’ endocytosis (Neef et al. 2014). CME is the main pathway of membrane retrieval for mild stimulation and proceeds at a constant rate; it represents the linear component of endocytosis following exocytosis of the RRP (Fig. 3a). This mechanism is not only inhibited by the clathrin-inhibitor pitstop-2 but also by disruption of dynamin 1 via pharmacological and genetic means (Neef et al. 2014). None of these manipulations seem to affect exocytosis. In contrast, a different study reported inhibition of sustained exocytosis by the presumptive dynamin inhibitor dynasore but did not investigate endocytic membrane retrieval (Duncker et al. 2013). Finally, when exocytosis exceeds three to four RRP equivalents, IHCs additionally recruit a faster mode of membrane retrieval, which proceeds with an exponential time course within a few seconds. It has been proposed to represent bulk endocytosis (Neef et al. 2014; Fig. 3a’) and, indeed, there is plenty of evidence for the invagination and fission of large stretches of plasma membrane in the vicinity of hair cell AZs (Lenzi et al. 2002; Frank et al. 2010; Pangršič et al. 2010; Kamin et al. 2014; Neef et al. 2014; Revelo et al. 2014). Both mechanisms seem to engage in different phases of release: CME supports vesicle cycling during mild stimulation but bulk endocytosis finally occurs after prolonged stimulation, providing a mechanism that assures the balance between exo- and endocytosis in IHCs and thus, assures high release rates (Neef et al. 2014).

But where does the retrieval of synaptic vesicle membrane take place and where and how are synaptic vesicles regenerated following membrane retrieval in hair cells? Does synaptic endocytosis comply with the apicobasal compartmentalization of the hair cell? At photoreceptor ribbons, a periactive zone, marked by the presence of endocytic proteins, was identified directly adjacent to the AZ area (in a range of 120–250 nm from the ribbon) using high-resolution and electron microscopy (Wahl et al. 2013). But does this also apply to IHC ribbons? At the resolution of confocal microscopy, a similar perisynaptic accumulation of endocytic proteins has, so far, not been found (Neef et al. 2014); however, future studies using nanoscopy will hopefully clarify this issue. Clearly, electron micrographs indicate that both bulk and CME endocytosis take place near the AZ (Siegel and Brownell 1986; Lenzi et al. 2002; Frank et al. 2010; Kamin et al. 2014; Neef et al. 2014). A radically different model of IHC endocytosis has been sketched based on life imaging of FM1-43 uptake into IHCs, whereby exocytosed membrane was postulated to move towards the IHC apex for endocytosis and recycling via the Golgi apparatus (Griesinger et al. 2002, 2005). Recent elaborative studies using various styryl dyes and more suitable fluorescent membrane markers lead us to reconsider this hypothesis. First, it was corroborated that many styryl dyes including FM1-43 permeate into the cytosol of IHCs via the mechanotransducer channels (Kamin et al. 2014; Revelo et al. 2014). Therefore, on the ultrastructural level, the photo-oxidation technique revealed a fuzzy dark diaminobenzidin (DAB) smear inside the cytosol in addition to the precipitate in membrane-bound organelles likely resulting from internalized FM1-43. The stimulation-induced endocytic uptake of FM1-43 could still be followed by observing an electron-dense precipitate within vesicular structures, which allows the determination of the presence or absence as well as the localization of stained structures under resting (Fig. 3b), stimulated (Fig. 3b’) and recovery (Fig. 3b”, b”’) conditions in serial 3D reconstructed IHCs (Kamin et al. 2014). At rest, endosome-like organelles were detected in the apex of the IHCs, whereas larger tubulo-cisternal organelles dominated at the nuclear region. At the basal region, only a few labeled structures were present. Stimulation massively increased the amount of basolateral membrane trafficking, reflected by the appearance of labeled small vesicles and endosome-like vacuoles; however, no changes in the apical and nuclear regions could be observed (Kamin et al. 2014; Fig. 3b’). Strikingly, the basolateral cisterns were replaced in the basal region by small, synaptic-like vesicles during a few minutes of recovery from stimulation, suggesting a highly efficient mechanism of vesicle regeneration from cisternal membranes internalized by bulk endocytosis. The combination of FM1-43 uptake and photo-oxidation therefore suggests that synaptic vesicle recycling takes place at the basal part, close to ribbons at least during synaptic activity (Kamin et al. 2014).

Recently, a novel membrane tracer, named membrane-binding fluorophore-cysteine-lysine-palmitoyl group (mCLING), which does not permeate the mechanotransducer channel, tightly binds biological membranes and can be fixed, has been developed (Revelo et al. 2014). In combination with super-resolution light microscopy (i.e., STED), the spatial organization and pathways of endocytosis in IHCs could be further investigated. In order to improve the spatial resolution of STED microscopy in the axial direction, thin sections of IHCs were imaged after embedding the organs of Corti in melamine, which maintains the fluorescence (Revelo et al. 2014). The apical, nuclear and basal regions under conditions of rest, stimulation and recovery from stimulation, were investigated and the uptake of mCLING monitored. In order to reveal the molecular identity of mCLING-labeled structures and thereby identify the respective endocytic pathways, samples were co-stained with different protein markers for the endoplasmic reticulum (ER) and Golgi as well as synaptic vesicle endo- and exocytosis. In these experiments, a strong correlation for basolateral mCLING localization with Vglut3, rab3 and otoferlin immunofluorescence was found. Otoferlin as well as syntaxin 16, a late endosomal marker, colocalized with apical and nuclear mCLING (Fig. 3c–e). Moreover, the lysosomal-associated membrane protein 1 (LAMP1) colocalized with mCLING in the apical region of the IHC. Stimulation led to a selective uptake of mCLING at the base of the IHC, corroborating the notion of local recycling of synaptic vesicles that was postulated based on electron microscopy and photo-oxidation (Kamin et al. 2014; Revelo et al. 2014). The local recycling hypothesis was further supported by the finding that exogenous Vglut1-pHluorin fluorescence not only transiently appeared at ribbon-type AZs but remained there for tens of seconds after stimulation (Neef et al. 2014; Revelo et al. 2014).

Finally, the mCLING experiments revealed large membranous organelles near synapses, which were replaced by small organelles a few minutes after stimulation, thereby providing direct evidence of bulk endocytosis and vesicle regeneration from the internalized plasma membrane. The association of otoferlin with all three putative membrane recycling pathways suggests a more general role of this protein in endocytosis. Otoferlin has recently been assigned a role in vesicle endocytosis due to its interaction with AP-2. Using a high-resolution liquid chromatography coupled with a mass spectrometry approach, multiple subunits of AP-2 were identified as interaction partners of otoferlin in the mammalian cochlea and the proposed interactions were biochemically confirmed by co-immunoprecipitation (Duncker et al. 2013). AP-2 plays a role in clathrin-mediated endocytosis via binding to clathrin-coated vesicles budding from the plasma membrane (Keyel et al. 2008; Boucrot et al. 2010) and has been shown to be expressed in IHCs (Duncker et al. 2013). Future work is required to clarify the role of AP-2 in hair cell endocytosis and the relevance of its interaction with otoferlin.

### Outlook

Recently, major progress has been made towards dissecting the molecular anatomy and physiology of hair cell ribbon synapses. This includes powerful single synapse techniques such as (1) patch-clamp of postsynaptic afferent terminals of SGNs, (2) high resolution -functional imaging of presynaptic IHC Ca^2+^ dynamics and membrane turnover, as well as (3) super-resolution light microscopy and electron tomography following high-pressure freezing. However, in order to investigate the release mechanisms of IHCs and firmly correlate structure and function, the development of new functional and morphological approaches is required. Functional and morphological analysis of single synapses will be necessary and some questions require reading out both pre- and postsynaptic properties at the same time. The commonly used K^+^ stimulation of cochlear tissue likely mimics strong physiological steady-state stimulation. But this stimulation does not provide the temporal resolution to allow the observation of the release kinetics at IHC ribbon synapses. Especially, knowledge about short-term plastic changes is lacking, since it is not possible to apply very short stimuli (i.e., millisecond range) and investigate the cells during and at defined times after stimulation. Therefore, approaches are needed that meet two requirements: (1) a precise stimulation protocol combined with (2) rapid immobilization of the sample, e.g., by using high-pressure freezing. One emerging tool that promises to fulfill these requirements is the combination of optogenetic stimulation with high-pressure freezing. This could involve the expression of a light-sensitive ion channel such as channelrhodopsin-2 (ChR-2) from the green algae *Chlamydomonas reinhardii* (Nagel et al. 2003) in hair cells and stimulation would ideally be performed within a chamber that should be mounted in a freezing machine in order to minimize the time delay before freezing. Such optogenetic investigations of synapses combined with electron microscopy have been emerging. Recently, synaptic recovery of motoneurons from *C. elegans* was analyzed using optogenetic stimulation in combination with high-pressure freezing (Kittelmann et al. 2013). Moreover, after a single light stimulus, docked vesicles fused along a broad AZ on *C. elegans* motoneurons expressing ChR-2. These vesicles were replenished with a time constant of about 2 s. Further, endocytosis occurred within 50 ms adjacent to the dense projection and after 1 s adjacent to adherens junctions (Watanabe et al. 2013a). Moreover, a study on optically stimulated cultured hippocampal neurons revealed an ultrafast endocytosis mechanism at central synapses (Watanabe et al. 2013b). These initial experiments indicate that optogenetics, in combination with high-pressure freezing (‘flash and freeze’; Watanabe et al. 2013b) and subsequent electron tomography, might provide sufficient resolution to study the ultrastructure of spatiotemporally defined functional states and thus provide a completely new view on the release mechanism of IHC ribbon synapses.
